# Relationships of Rheumatoid Factor with Thickness of Retina and Choroid in Subjects without Ocular Symptoms Using Swept-Source Optical Coherence Tomography

**DOI:** 10.1155/2021/5547533

**Published:** 2021-03-24

**Authors:** Qingjian Li, Yiwen Qian, Sennan Xu, Minjie Zhang, Xu Liang, Xin Che, Jing Jiang, Zuguo Liu, Yu Zhang, Yan Liu, Zhiliang Wang

**Affiliations:** ^1^Eye Institute of Xiamen University; School of Medicine, Xiamen University, Department of Ophthalmology, Xiang'an Hospital, Xiamen University, Xiamen, Fujian, China; ^2^Department of Ophthalmology, Huashan Hospital, Fudan University, Shanghai, China

## Abstract

**Purpose:**

Researches have confirmed that the retinal and choroidal thickness in patients with autoimmune disease-associated uveitis displays significant changes. However, the relationships between rheumatoid factor (RF) and thickness of the retina and choroid in individuals without ocular manifestations remain unclear. The aim of this study is to assess the associations of RF with retinal and choroidal thickness.

**Methods:**

The individuals enrolled in the cross-sectional research received full ocular examinations. The participants were classified as the RF (+) group (RF ≥ 15.0 IU/ml) and the RF (−) group (RF < 15.0 IU/ml) according to the serum RF titers. The thickness of the retina and choroid was measured by swept-source optical coherence tomography (SS-OCT).

**Results:**

The study covered 65 right eyes of 65 individuals that are RF-positive and 130 right eyes of 130 age- and sex-matched individuals that are RF-negative. The RF (+) group showed decreased choroidal thickness that achieved statistical significance only in the outer inferior and outer temporal sectors, as compared to the RF (−) group. There was no statistically significant difference regarding the retinal thickness between the two groups. Pearson's correlation analysis revealed that the RF was significantly negatively related to the choroidal thickness in all areas. However, there was no significant correlation between the RF and the retinal thickness.

**Conclusions:**

Serum RF titers are closely linked with choroidal thickness before the emergence of ocular symptoms. Research into the relationships may improve our understanding of the role of serum RF in the pathogenesis of uveitis.

## 1. Introduction

Rheumatoid factor (RF) is a series of autoantibodies with various isotypes and affinities, directed against the fragment-crystallizable (Fc) portion of immunoglobulin G (IgG) [1–3]. Among the isotypes primarily including IgM, IgA, and IgG, the IgM is commonly mentioned due to the efficiency in agglutination reactions, while other isotypes are rarely found [1, 2]. In contrast to what the name implies, RF is present not only in rheumatoid arthritis but also in a variety of diseases including other rheumatic and nonrheumatic disorders. Some people have elevated RF before the symptomatic abnormality. Also, it is present in 1-16% of general population without inflammatory diseases [4–6]. The presence, titers, and isotypes of RF have great implications for the diagnosis and prognosis of autoimmune diseases [1].

The eye is one of the most susceptible organs, in terms of inflammatory infiltration, metabolic disturbance, and vascular abnormality. Severe ocular inflammation involving the entire globe from the anterior segment to the posterior segment can be caused by the dysregulation of the immune system. However, effective treatment of patients affected by ocular inflammation remains challenging for numerous ophthalmologists. Early identification of signs and accurate diagnosis can offer breakthrough approaches to overcome the challenges. Recently, swept-source optical coherence tomography (SS-OCT) has been developed as a new technology to meet the increasing demand for fast and reliable diagnosis of ocular fundus diseases.

Homeostasis of the retina and choroid is essential for normal visual function. The thickness of the retina and choroid can be affected by both systemic diseases and physiological conditions [7, 8]. Several studies have pointed out that the thickness of the retina and choroid in patients with autoimmune disease-associated uveitis displays significant changes on OCT [9–14]. However, no research has evaluated the associations between serum RF and thickness of the retina and choroid before the emergence of ocular symptoms. Research into the relationships of serum RF with thickness of the retina and choroid may improve our understanding of the role of RF in subjects with uveitis. The aim of this study is to evaluate whether RF titers in individuals without ocular manifestations are related to thickness of the retina and choroid by SS-OCT.

## 2. Methods

### 2.1. Study Population

The cross-sectional research was carried out at Huashan Hospital of Fudan University from February 2019 to December 2019, in conformity to the tenets of the Declaration of Helsinki. Ethical approval was achieved from the Institutional Review Board of Huashan Hospital. Informed consent was signed by all participants enrolled in the research. All individuals received full ocular examinations like best-corrected visual acuity (BCVA), refractive error, intraocular pressure (IOP), slit-lamp microscope, funduscopy, and SS-OCT scan. Blood samples were collected between 8:00 and 10:00 after an eight-hour overnight fast. The normal reference value of serum RF was less than 15.0 IU/ml. The participants were grouped into the RF (+) group (RF ≥ 15.0 IU/ml) and the RF (−) group (RF < 15.0 IU/ml) according to the RF titers. Each right eye was included in the analysis, as a single experiment unit. Two participants that are RF-negative were paired with one participant that is RF-positive to improve the reliability.

### 2.2. Inclusion Criteria

The inclusion criteria were as follows: (a) age 18-69 years; (b) 10 ≤ IOP ≤ 21 mmHg; (c) BCVA ≥ 20/25 Snellen; (d) −6 < spherical equivalent < +6 diopters; (e) no history of ocular abnormalities like uveitis, glaucoma, and age-related macular degeneration; (f) no history of ocular surgeries; (g) no history of diabetes mellitus, hypertension, and, thyroid diseases; and (h) no history of corticosteroid therapy during the past 3 months.

### 2.3. Swept-Source Optical Coherence Tomography Imaging

SS-OCT version 9.31 (DRI OCT-1 Atlantis, Topcon Co., Tokyo, Japan) used a longer wavelength (1050 nm) to reduce the light attenuation on the choroid [15]. It could provide more clear images of the fundus with a scan speed of 100,000 A scans/s. Twelve equidistant radial line scans with a length of 9 mm were centered on the fovea of the macula. The distances between the internal limiting membrane (ILM) and retinal pigment epithelium (RPE) and between RPE and chorioscleral interface (CSI) were considered retinal thickness and choroidal thickness, respectively. Thickness map in accordance with the standard Early Treatment Diabetic Retinopathy Study (ETDRS) grid was created automatically, which consisted of 3 concentric circular areas (diameter: 1, 3, and 6 mm, separately) with nine independent sectors (center, inner superior, inner nasal, inner inferior, inner temporal, outer superior, outer nasal, outer inferior, and outer temporal). For every OCT scan, the ETDRS grid and the segmented lines (ILM, RPE, and CSI) were checked manually and adjusted if needed to avoid possible errors. All OCT scans in this research were completed at 8-10 am to avoid diurnal variations in thickness of the retina and choroid [16]. An experienced ophthalmologist captured a high-quality imaging for each right eye without knowing the RF levels.

### 2.4. Statistical Analysis

Statistical analysis was conducted with SPSS version 24.0 (SPSS, IBM Inc., Chicago, IL, USA). Data were presented as mean ± standard deviation (SD) for continuous variables and frequencies (percentages) for categorical variables. Comparisons between groups were performed with Student's *t*-test for continuous data and chi-square test for categorical data. The relationships between variables were evaluated with Pearson's correlation analysis. Statistical significance was defined as two-tailed *P* < 0.05.

## 3. Results

The study covered 65 right eyes of 65 individuals that are RF-positive and 130 right eyes of 130 age- and sex-matched individuals that are RF-negative.  Table 1 summarized the demographic characteristics. The average RF titer was 97.97 ± 140.49 mg/l in the RF (+) group and 6.06 ± 3.53 mg/l in the RF (−) group. The male-to-female ratio was 38 : 27 in the RF (+) group and 76 : 54 in the RF (−) group. The average age was 48.77 years (range, 21–68 years) in the two groups.

**Figure 1:**
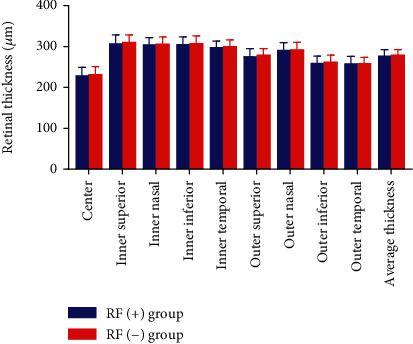
The retinal thickness by group. There was no statistically significant difference regarding the retinal thickness between the RF (+) group and the RF (−) group.

**Figure 2:**
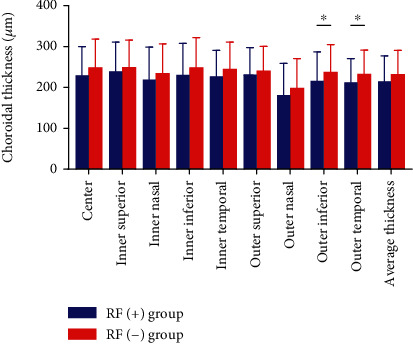
The choroidal thickness by group. The RF (+) group showed decreased choroidal thickness that achieved statistical significance only in the outer inferior and outer temporal sectors, as compared to the RF (−) group (^∗^*P* < 0.05).

In all sectors of the ETDRS grid, no statistically significant difference between the RF (+) group and the RF (−) group was observed regarding the retinal thickness (Table 2 and Figure 1). Data showed that the choroidal thickness in the subjects that are RF-positive was significantly thinner than that in the subjects that are RF-negative only in the outer inferior and outer temporal sectors of the ETDRS grid (Table 3 and Figure 2).

Relationships of serum RF with retinal and choroidal thickness are shown in Table 4 and 5, respectively. Pearson's correlation analysis revealed that there was no significant correlation between the RF and the retinal thickness in all sectors of the EDTRS grid. In contrast, the RF was significantly negatively correlated with the choroidal thickness in all areas.

## 4. Discussion

In this research, we, for the first time, assessed the correlations between RF levels and retinal and choroidal thickness by SS-OCT in subjects without ocular manifestations. The outcomes showed that the choroidal thickness in the subjects that are RF-positive was significantly thinner in the outer inferior and outer temporal sectors of the ETDRS grid. There was no statistically significant difference regarding the retinal thickness between the two groups. Pearson's correlation analysis revealed that the RF was significantly negatively correlated with the choroidal thickness in all areas. However, there was no significant correlation between the RF and the retinal thickness. This means that serum RF is closely related to choroidal thickness in subjects without ocular manifestations.

As one of the landmarks in the visualization of the retina and choroid, the SS-OCT could precisely recognize the CSI in the subject with thick choroid due to the strong penetrating power through the RPE [15]. The SS-OCT displayed an excellent accuracy in detecting the CSI [17, 18]. In other OCT types, the measurements could be affected by the local choroidal thinning or thickening, because the CSI was irregularly shaped in some people [19, 20]. Furthermore, there was a possibility of human error in manual measurement. These limitations could be overcome by SS-OCT [21, 22]. In the research, the thickness of the retina and choroid was averaged automatically by the SS-OCT device.

The serum RF levels were known to be elevated in most rheumatic diseases, such as rheumatoid arthritis, Sjogren syndrome, connective tissue diseases, and systemic lupus erythematosus [23]. Certain antigens could initiate activation of B cells and induced secretion of RF. The RF involved in the immune complex formation might lead to complement activation and recruitment of inflammatory cells including lymphocytes, macrophages, and neutrophils. The constant stimulation of the immune system could result in a chronic inflammatory state [24, 25]. Balmforth et al. [26] revealed that choroidal thinning was linked with inflammation in chronic kidney disease. Fang et al. [27] demonstrated that C-reactive protein was significantly negatively related to choroidal thickness before the emergence of ocular symptoms. In patients with uveitis, the choroidal thickness displayed significant changes on OCT [12, 13]. Yesilirmak et al. [12] found that choroidal thickness was significantly thinner in patients with Behcet's disease-associated uveitis in end-stage phase compared with that in normal subjects. Park et al. [13] also discovered that choroidal thickness decreased over time in patients with Behcet's disease-associated uveitis and the mean change rate was greater than that in controls (-7.2 versus -2.0 *μ*m/year; *P* < 0.001). These may help explain the negative correlation between RF and choroidal thickness in our study. The second possible cause is RF-related vascular abnormities. RF was one of the risk factors for atherosclerosis which was related to decreased vessel density and blood flow area in the choroid [28–31]. Also, patients with some nonrheumatic diseases, especially chronic infections like subacute infective endocarditis, hepatitis B, and tuberculosis frequently had RF elevation [6, 32]. The chronic infections may result in an obvious decrease in the choroidal thickness in the subjects that are RF-positive.

More than 70% of ocular blood flow was in the choroid, while approximately 4% was in the retina [33, 34]. The retina and choroid differed substantially with respect to blood flow regulation; the latter employed autonomic regulation instead of the autoregulation employed by the former [35–37]. Furthermore, the blood-retina barrier prevented harmful substances from entering the eye. The differences in blood flow and barrier function may clarify why the choroid is more affected by RF than the retina. Consistent with our findings, a study by Basarir et al. [38] concluded that the retinal thickness was not affected in HLA-B27-positive ankylosing spondylitis patients with anterior uveitis. Nevertheless, we consider, with the persistence of elevated IF, the retinal thickness and the choroidal thickness in the other sectors will be affected by inflammatory infiltration.

The RF elevation would lead to a higher risk of individuals having rheumatoid arthritis. Also, the patients that are RF-positive experienced more aggressive and erosive joint diseases and extraarticular manifestations such as rheumatoid nodules and vasculitis than those that are RF-negative [39]. Our research demonstrated that RF titers were related to choroidal thickness before the emergence of ocular symptoms. A decreased choroidal thickness might cause lower choriocapillaris perfusion that might result in ischemia of the outer retina [40]. So the decreased choroidal thickness may be an important clue to prevent specific eye diseases. Early recognition together with a correct diagnosis and treatment of the subjects with increased RF levels could reduce the incidence of some severe ocular complications. The collaboration of ophthalmologists, immunologists, and rheumatologists is essential for the successful care of patients with uveitis.

## 5. Conclusions

In summary, serum RF levels are closely linked with choroidal thickness before the emergence of ocular symptoms. Research into the relationships may improve our understanding of the role of serum RF in the pathogenesis of uveitis.

## Figures and Tables

**Table 1:** Demographic characteristics.

Parameter	RF (+) group	RF (−) group	*P* value
Subject, *n*	65	130	—
Eye, *n*	65	130	—
Sex, *n* (%)			1.000^a^
Male	38 (58.5)	76 (58.5)	
Female	27 (41.5)	54 (41.5)	
Age (year)	48.77 ± 9.20	48.77 ± 9.16	1.000^b^
Range	21–68	21–68	
RF (IU/ml)	97.97 ± 140.49	6.06 ± 3.53	<0.001^b^

RF: rheumatoid factors; ^a^chi-square test; ^b^*t*-test.

**Table 2:** The retinal thickness by group.

Retinal thickness	RF (+) group	RF (−) group	*P* value
	*n* = 65	*n* = 130	
Center (*μ*m)	230.27 ± 18.40	231.92 ± 19.18	0. 567^b^
Inner superior (*μ*m)	308.38 ± 20.18	310.79 ± 17.46	0. 390^b^
Inner nasal (*μ*m)	305.78 ± 16.06	306.43 ± 17.22	0. 798^b^
Inner inferior (*μ*m)	306.44 ± 17.41	307.84 ± 18.22	0. 609^b^
Inner temporal (*μ*m)	299.11 ± 14.18	300.16 ± 16.16	0. 658^b^
Outer superior (*μ*m)	276.38 ± 18.61	279.41 ± 15.23	0. 227^b^
Outer nasal (*μ*m)	292.75 ± 16.74	292.41 ± 17.61	0. 895^b^
Outer inferior (*μ*m)	261.01 ± 15.39	262.47 ± 16.41	0. 551^b^
Outer temporal (*μ*m)	259.69 ± 16.20	259.10 ± 14.46	0. 803^b^
Average thickness (*μ*m)	278.51 ± 14.26	279.52 ± 13.17	0. 623^b^

^b^ *t*-test.

**Table 3:** The choroidal thickness by group.

Choroidal thickness	RF (+) group	RF (−) group	*P* value
	*n* = 65	*n* = 130	
Center (*μ*m)	230.55 ± 69.58	248.34 ± 69.71	0. 095^b^
Inner superior (*μ*m)	240.14 ± 70.50	249.29 ± 66.31	0. 375^b^
Inner nasal (*μ*m)	219.76 ± 78.33	234.66 ± 71.41	0. 185^b^
Inner inferior (*μ*m)	231.11 ± 76.32	248.71 ± 73.28	0. 121^b^
Inner temporal (*μ*m)	227.98 ± 62.71	244.72 ± 66.12	0. 092^b^
Outer superior (*μ*m)	232.01 ± 65.52	240.83 ± 59.50	0. 347^b^
Outer nasal (*μ*m)	181.84 ± 76.85	198.13 ± 72.48	0. 149^b^
Outer inferior (*μ*m)	216.83 ± 70.08	237.53 ± 67.52	0. 048^b^
Outer temporal (*μ*m)	213.14 ± 57.39	232.74 ± 58.72	0. 028^b^
Average thickness (*μ*m)	215.60 ± 61.86	231.61 ± 59.30	0. 081^b^

^b^t-test.

**Table 4:** The relationship of serum RF with retinal thickness.

Parameter	Center	Inner superior	Inner nasal	Inner inferior	Inner temporal	Outer superior	Outer nasal	Outer inferior	Outer temporal	Average thickness
RF										
*r* value	-0.015	-0.046	-0.022	-0.013	-0.033	-0.078	-0.032	-0.080	-0.057	-0.064
*P* value	0.835^c^	0.525^c^	0.759^c^	0.860^c^	0.644^c^	0.280^c^	0.656^c^	0.267^c^	0.432^c^	0.377^c^

RF: rheumatoid factor; ^c^Pearson's correlation analysis.

**Table 5:** The relationship of serum RF with choroidal thickness.

Parameter	Center	Inner superior	Inner nasal	Inner inferior	Inner temporal	Outer superior	Outer nasal	Outer inferior	Outer temporal	Average thickness
RF										
*r* value	-0.261	-0.220	-0.245	-0.201	-0.236	-0.187	-0.214	-0.203	-0.209	-0.233
*P* value	<0.001^c^	0.002^c^	0.001^c^	0.005^c^	0.001^c^	0.009^c^	0.003^c^	0.004^c^	0.003^c^	0.001^c^

RF: rheumatoid factor; ^c^Pearson's correlation analysis.

## Data Availability

The data sets used and/or analyzed during the current study are available from the corresponding author on reasonable request.
